# Effect of Hypericin-Mediated Photodynamic Therapy on the Secretion of Soluble TNF Receptors by Oral Cancer Cells

**DOI:** 10.3390/pharmaceutics15041279

**Published:** 2023-04-19

**Authors:** Marcin Olek, Agnieszka Machorowska-Pieniążek, Zenon P. Czuba, Grzegorz Cieślar, Aleksandra Kawczyk-Krupka

**Affiliations:** 1 Doctoral School of Medical University of Silesia, 40-055 Katowice, Poland; 2 Department of Orthodontics, Faculty of Medical Sciences in Zabrze, Medical University of Silesia, 40-055 Katowice, Poland; apieniazek@sum.edu.pl; 3 Department of Microbiology and Immunology, Faculty of Medical Sciences in Zabrze, Medical University of Silesia, 40-055 Katowice, Poland; zczuba@sum.edu.pl; 4 Department of Internal Diseases, Angiology and Physical Medicine, Center for Laser Diagnostics and Therapy, Faculty of Medical Sciences in Zabrze, Medical University of Silesia, 40-055 Katowice, Poland; cieslar1@tlen.pl (G.C.); akawczyk@gmail.com (A.K.-K.)

**Keywords:** photodynamic therapy, oral cancer, hypericin, tumor necrosis factor receptors, plant compounds

## Abstract

Squamous cell carcinoma is the most common cancer of the head and neck region. In addition to the classic surgical treatment method, alternative therapy methods are sought. One such method is photodynamic therapy (PDT). In addition to the direct cytotoxic effect, it is essential to determine the effect of PDT on persistent tumor cells. The study used the SCC-25 oral squamous cell carcinoma (OSCC) cell line and the HGF-1 healthy gingival fibroblast line. A compound of natural origin—hypericin (HY)—was used as a photosensitizer (PS) at concentrations of 0–1 µM. After two hours of incubation with the PS, the cells were irradiated with light doses of 0–20 J/cm^2^. The 3-[4,5-dimethylthiazol-2-yl]-2,5-diphenyltetrazolium bromide (MTT) test was used to determine sublethal doses of PDT. Cell supernatants subjected to sublethal PDT were assessed for soluble tumor necrosis alpha receptors (sTNF-R1, sTNF-R2). The phototoxic effect was observed starting with a light dose of 5 J/cm^2^ and amplified with the increase in HY concentration and light dose. A statistically significant increase in sTNF-R1 secretion by SCC-25 cells was demonstrated after the PDT with 0.5 µM HY and irradiation with 2 J/cm^2^ (sTNF-R1 concentration = 189.19 pg/mL ± 2.60) compared to the control without HY and irradiated with the same dose of light (sTNF-R1 concentration = 108.94 pg/mL ± 0.99). The baseline production of sTNF-R1 was lower for HGF-1 than for SCC-25, and secretion was not affected by the PDT. The PDT had no effect on the sTNF-R2 production in the SCC-25 or HGF-1 lines.

## 1. Introduction

Oral cancer belongs to the group of head and neck cancers (HNCs), and its most common origin is epithelial cells, accounting for 90% of cases, hence the name oral squamous cell carcinoma (OSCC) [1]. OSCC is a malignant neoplasm within the buccal mucosa, an inner surface of the lip, alveolar processes, tongue, retromolar triangle, oral cavity floor, and palate. Significant risk factors include cigarette smoking, alcohol consumption, chewing betel leaves and areca nuts, and human papillomavirus (HPV) infection [1,2,3]. Moreover, the role of poor oral hygiene and pathogens causing periodontitis as a factor increasing the risk of OSCC are emphasized [4]. The coexistence of risk factors multiplies the risk of OSCC; for example, the simultaneous consumption of alcohol and smoking increases the chance of developing cancer 35 times [2]. In 2020, 377,713 new oral cavity and lip cancer cases were recorded, of which about 70% were male, and 177,757 people died [5]. The standard of treatment for OSCC is resection of the tumor within healthy tissues with possible neck dissection in the presence of lymph node metastases. In the case of disseminated metastases in lymph nodes or the inability to perform radical resection, adjuvant radiotherapy or chemotherapy is used [6]. In selected cases of OSCC, chemotherapy can be used. The observed complications depend on the drug used, and the most common are systemic toxicity, neuropathy, hair loss, gastric problems, opportunistic infection, mucositis, pain, xerostomia, and dysphagia [7,8]. Despite reconstructive surgery and rehabilitation, resection of the tumor is often associated with complications, such as speech, chewing, and swallowing disorders, and causes aesthetic problems for patients [9]. In turn, the complications of radiotherapy include pain, xerostomia, tissue fibrosis, mucositis, opportunistic infections, increased susceptibility to caries, and periodontal disease [10]. The listed complications of classical OSCC therapy have a negative impact on the quality of life [11,12]. The reasons mentioned above lead to the search for new alternative treatment methods.

One of the alternative methods of treating precancerous lesions and OSCC is photodynamic therapy (PDT) [13,14]. Three components are needed in PDT: a photoactive agent called a photosensitizer (PS), oxygen found in the tissues, and a light source of the appropriate wavelength [15]. The advantages of PDT are its low invasiveness and selectivity of action. Its applicability depends on the stage of cancer. An important factor affecting the effectiveness of PDT is the thickness of the tumor infiltration, as the effect of the therapy relies on the penetration of light into the tissues. Therefore, PDT can be used in the early stages of cancer or superficial recurrence [13]. Hamdoon et al. determined the average thickness of oral lesions. Severe dysplasia was characterized by an average epithelial thickness of 484 μm, carcinoma in situ 570 μm, and microinvasive SCC 650 μm [16]. In addition, it is possible to use PDT as adjuvant therapy to classical surgical treatment and palliative treatment of advanced OSCC [17]. In addition to the cytotoxic effect, an important mechanism of PDT action is the immunomodulatory effect and the destruction of blood vessels supplying the tumor [18]. Preclinical and clinical trials of the use of PDT in treating OSCC and oral precancerous changes have been conducted, and their results encourage further work on this type of therapy. Preclinical studies on animals have shown inhibition of tumor growth or complete response, increased life expectancy, and no or minor side effects of PDT [19]. For treating oral potentially malignant disorders (OPMDs), 60% of cases have been reported to have a complete response and 90% to have a partial response. PDT is effective in dysplasia, carcinoma in situ, and actinic cheilitis [19]. It was found that PDT was more effective after administration of the photosensitizer systemically than topically, which may be related to the increased keratosis associated with OPMDs [20,21]. PDT is used as an alternative for surface changes not exceeding 5 mm. The procedure is minimally invasive. It can be used repeatedly without causing damage to oral tissues. In the case of advanced and inoperable cancers, it can improve a patient’s quality of life [22].

Many compounds of natural and synthetic origin can be used as PSs. Hypericin (HY), isolated from the Hypericum Perforatum plant, is one of the most potent photosensitizers of a natural origin. It is characterized by low solubility in water. In order to increase solubility and avoid the formation of aggregates, it is necessary to modify it with other compounds. Regarding the optical properties of HY, the absorbance of electromagnetic radiation is 500 to 620 nm of visible light, with maximum absorption at 595 nm [23,24]. The light of such a wavelength is characterized by lower penetration into tissues, which allows the treatment of only the early stages of cancer [25]. Due to the absorption range of hypericin, its use in PDT will only be useful for eradicating thin layers of malignant cell types, especially in the early stages of oral cancer, carcinoma in situ, or precancerous lesions. On the other hand, shorter wavelengths, characterized by short optical penetration, reduce the overall treatment depth. This can be a positive feature in the treatment of easily accessible superficial lesions, as it increases PS activation at shallower depths, thus avoiding damage to underlying healthy tissues [26].

It is important to understand the role of PDT’s immunomodulatory effect, including the production of cytokines by persistent cells. Tumor necrosis factor alpha (TNF-α) is a cytokine that plays a vital role in the inflammatory process as a potent proinflammatory agent [27]. Inflammation can be an important element leading to carcinogenesis, as in the case of oral cancer [28]. TNF-α has a pluripotent effect. Depending on the concentration, the receptor with which it binds, and the metabolic state of the target cell, it can cause a proapoptotic or antiapoptotic effect [29]. There are two receptors for TNF-α; TNF-R1 is found on most nuclear cells, while TNF-R2 is found mainly on the surface of the immune system cells, endothelial cells, and fibroblasts [30]. Through its receptors, TNF-α affects the arachidonic acid cascade, causing an increase in the concentration of intracellular free radicals, leading to cell apoptosis. TNF-α creates a proinflammatory microenvironment. It may exert an effect on mesenchymal stem cells, increasing their anticancer activity [31]. Both TNF-α and its receptors can occur in transmembrane and soluble form, and their release is the responsibility of the tumor necrosis factor-alpha converting enzyme (TACE). As a result of the proteolytic action of the enzyme, soluble TNF-α (sTNF-α) and soluble receptors are formed (sTNF-R1, sTNF-R2). The free forms of the receptors may weaken the effect of TNF-alpha by competitively binding to membrane receptors, or they may prolong its activity by stabilizing it and protecting it against degeneration [27].

## 2. Materials and Methods

### 2.1. Chemicals

HY, dimethyl sulfoxide (DMSO), MTT (3-[4,5-dimethylthiazol-2-yl]-2,5-diphenyltetrazolium bromide), and hydrocortisone were purchased from Sigma–Aldrich (St. Louis, MO, USA). Dulbecco’s Modified Eagle’s Medium (DMEM), DMEM: F-12, inactivated fetal bovine serum (FBS), and trypsin (0.23%)-ethylenediaminetetraacetic acid (EDTA) (0.53 mM) were obtained from ATCC (Manassas, VA, USA). Dulbecco’s phosphate-buffered saline (DPBS) without calcium and magnesium ions was obtained from PAA. Bio-Plex Pro Assays were obtained from BIO-RAD Laboratories, Inc., (Hercules, CA, USA). All other chemicals were of analytical grade or purer.

### 2.2. Cell Cultures

This study used two human cell lines from ATCC (American Type Cell Culture—ATCC LGC Limited. Queens Road, Teddington, Middlesex, TW11 0LY, UK). The first one—SCC-25 (ATCC CRL-1628)—is OSCC isolated from the tongue of a 70-year-old male. The cells of the second line—HGF-1 (ATCC CRL-2014)—are normal gingival fibroblasts from a 28-year-old male. The cells were cultured according to the manufacturer’s recommendations. The SCC-25 line was cultured in DMEM: F12 medium supplemented with 400 ng/mL hydrocortisone and 10% FBS. In turn, HGF-1 in DMEM medium with an added FBS to a final concentration of 10%. Both cell lines were grown in 75 cm^3^ flasks at 37 °C and an atmosphere containing 5% CO_2_. The medium was renewed 2–3 times a week, and the cells were passaged before obtaining 80% monolayer confluence. The cells were harvested using 0.25% (*w*/*v*) Trypsin-0.53 mM EDTA solution. Then the culture media were added to complete the trypsinization process. The cells were later diluted in culture media to a final concentration of 1 × 10^5^/mL for SCC-25 and 1 × 10^4^/mL for HGF-1. For further stages of the research, the cells were seeded on a 96-well plate in equal amounts of 200 µL per well. To obtain cell adherence, they were left for 24 h in an incubator at 37 °C and in an atmosphere containing 5% CO_2_ and constant humidity.

### 2.3. Fluorescence Microscopy

A 1 mM stock solution was obtained by dissolving HY in DMSO, and working solutions were obtained by diluting stock solution in the appropriate cell culture media. The final concentration of DMSO was ≤0.01%. Adhered SCC-25 and HGF-1 cells were incubated with HY at concentrations of 0 µM, 0.25 µM, 0.5 µM, and 1 µM for two hours. The presence of HY in the cells was confirmed using an Olympus IX51 inverted research microscope (Olympus Inc., Tokyo, Japan) with a color view camera and Cell F version 2.6 (Soft Imaging System GmbH) software for recording and processing microscopic images. A fluorescein isothiocyanate (FITC) filter was used at 200× magnification to obtain pictures.

### 2.4. Photodynamic Therapy

The SCC-25 and HGF-1 cells, after 24 h of adherence, were incubated for 2 h with HY at concentrations of 0 µM, 0.25 µM, 0.5 µM, and 1 µM. The experiment was carried out without direct access to light from the moment of adding HY. After incubation with the PS, the cells were rinsed with PBS without calcium and magnesium ions, and the culture medium was replaced. Then the cells were exposed to visible light VIS (450–720 nm) from an incoherent light source (TP-1 (Cosmedico Medizintechnik Gm-bH, Schwenningen, Germany)) as PDT. The orange and infrared light filters allowed the passage of 580–720 nm wavelengths. The light doses of 0 J/cm^2^, 1 J/cm^2^, 2 J/cm^2^, 5 J/cm^2^, 10 J/cm^2^, and 20 J/cm^2^ were used for the experiment. The controller automatically counted the exposure time. The plate containing the dark control was not exposed to light, while in the case of plates exposed to irradiation, the exposure time increased with an increasing light dose. The study used a non-laser light source for the tested plate to obtain a uniform light dose, which is possible thanks to the wide illumination field [32]. A double water filter was used to avoid the heating effect. After irradiation, the cells were incubated for 24 h in the dark. With the water filters used, the fluence rate was 35 mW/cm^2^.

### 2.5. MTT Assay for Cytotoxicity Evaluation

After 24 h of incubation, supernatants were collected, and fresh medium with MTT (3-(4,5-dimethylthiazol-2-yl)-2,5-diphenyltetrazolium bromide) at a concentration of 0.5 mg/mL was added to the cells. The cells were then further incubated in the dark for four hours. Only living cells can metabolize soluble MTT to insoluble formazan crystals; this test indicates the activity of mitochondrial dehydrogenases. After the incubation, the medium with unreduced MTT was discarded, and DMSO was added to dissolve the formazan. After adding DMSO, the plates were placed in a shaker for 10 min to dissolve the crystals completely. The solution was then transferred to a polypropylene plate, and the absorbance was determined spectrophotometrically using a microplate reader (ELx 800, Bio-Tek Instruments Inc., Winooski, VT, USA) at a wavelength of 550 nm.

### 2.6. sTNF-R1 and sTNF-R2 Concentration Measurement

The samples for which PDT was sublethal were used for the study. The assessment of cytokine secretion by cells exposed to therapy at the highest doses not yet causing a cytotoxic effect allows the assessment of the immunomodulatory effect of PDT. That protocol has been used in previous studies [33,34,35,36]. The levels of sTNF-R1 and sTNF-R2 were determined in the collected supernatants using the Bio-Plex Pro Assay. All procedures were carried out strictly according to the manufacturer’s instructions. This method was based on magnetic polystyrene beads coated with monoclonal antibodies directed against the detected biomarkers. After the addition of buffer-diluted samples and incubation, a series of washes were performed to remove unbound proteins. A biotinylated detection antibody was then added to the wells and bound to other epitopes of the biomarkers under study to form a sandwich complex. After another series of washes and removal of unbound antibodies, streptavidin–phycoerythrin (SA-PE) conjugate was added. The bound phycoerythrin served as a fluorescence detector. After another washing and removal of the unbound SA-PE complex, the beads coated with the final detection complex were suspended in the assay buffer. The data were obtained using the Bio-Plex 200 System. In the reader, each bead was illuminated simultaneously with a red (635 nm) and green (532 nm) laser. The red light was used to identify beads by illuminating fluorescent dyes on their surface, while the green light excited PE fluorescence. The data were processed in Bio-Plex Manager Software version 6.0. The value of the tested biomarkers was read from the standard curves obtained using the standards provided by the manufacturer. The multiplex assay method is used in preclinical and clinical studies to determine the level of cytokines [37,38,39].

### 2.7. Statistical Analysis

All measured values are presented as means  ±  SE (standard error). The normality of the distribution was checked by the Shapiro–Wilk test. The two-tailed Student’s *t*-test was used to compare the control and the rest of the groups in the MTT metabolic activity assay. The Bio-Plex results did not show a normal distribution. Therefore, the Kruskal–Wallis analysis of variance (ANOVA) was used. A post hoc analysis was performed for the Kruskal–Wallis ANOVA using a multiple comparison (Dunn) test. Statistica version 13 (TIBCO Software Inc., Palo Alto, CA, USA, 2017) and Excel (Microsoft 365, 2303 version) software were used to analyze the results. Values of *p*  <  0.05 were considered statistically significant. All experiments were repeated four times, n = 4.

## 3. Results

### 3.1. Fluorescence Microscopy

**Figure 1:**
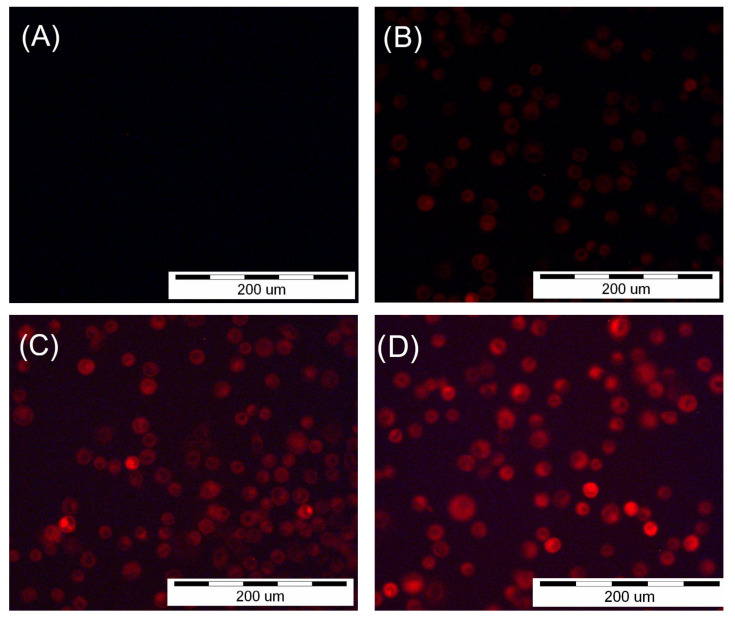
Images of the SCC-25 cells were obtained using an inverted fluorescence microscope after two-hour incubation with HY. (**A**) No fluorescence after incubation with 0 µM HY solution. (**B**) Fluorescence after incubation with 0.25 µM HY solution. (**C**) Fluorescence after incubation with 0.5 µM HY solution. (**D**) Fluorescence after incubation with 1 µM HY solution.

**Figure 2:**
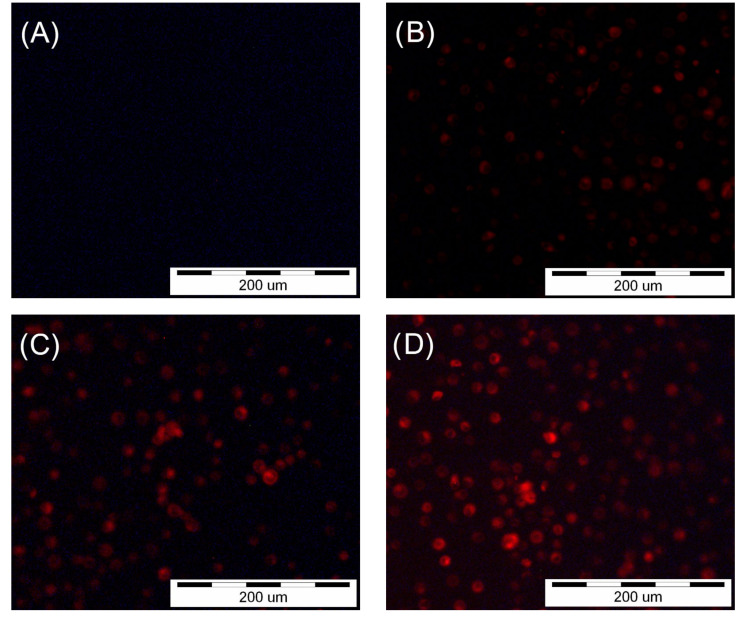
Images of the HGF-1 cells were obtained using an inverted fluorescence microscope after two-hour incubation with HY. (**A**) No fluorescence after incubation with 0 µM HY solution. (**B**) Fluorescence after incubation with 0.25 µM HY solution. (**C**) Fluorescence after incubation with 0.5 µM HY solution. (**D**) Fluorescence after incubation with 1 µM HY solution.

Observation of the SCC-25 and HGF-1 cells with a fluorescence microscope showed HY uptake by both cell lines (Figure 1 and Figure 2). The images showed a lack of fluorescence in the control groups not treated with HY. In the case of cells incubated with HY, an increase in fluorescence was seen with increasing concentrations of HY in the culture medium for both SCC-25 and HGF-1.

### 3.2. MTT Cytotoxicity Assay

**Figure 3:**
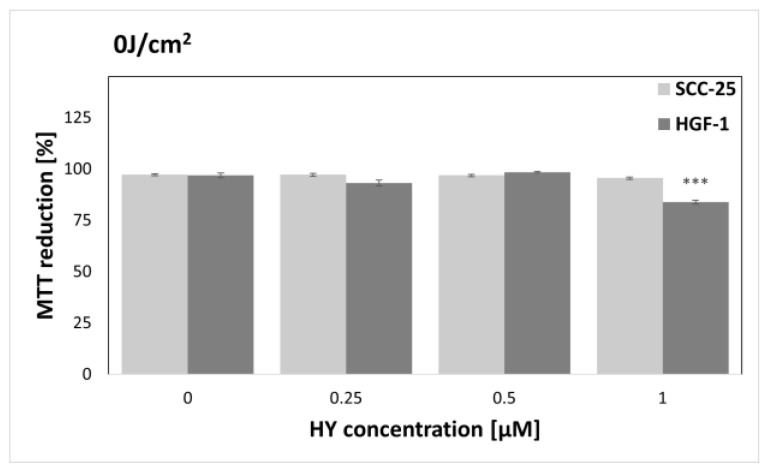
MTT reduction in the SCC-25 and HGF-1 lines for different HY concentrations: 0 μM, 0.25 μM, 0.5 μM, and 1 μM and light dose 0 J/cm^2^. The values represent the means ± SE. *** *p* < 0.01.

**Figure 4:**
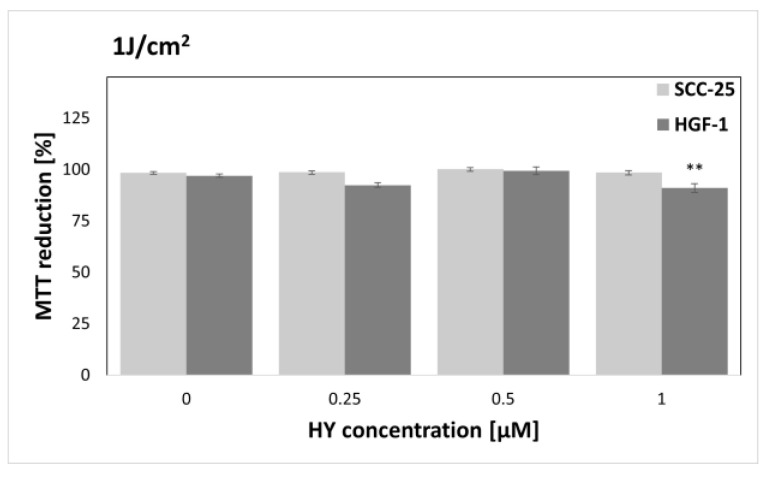
MTT reduction in the SCC-25 and HGF-1 lines for different HY concentrations: 0 μM, 0.25 μM, 0.5 μM, and 1 μM and light dose 1 J/cm^2^. The values represent the means ± SE. ** *p* < 0.05.

**Figure 5:**
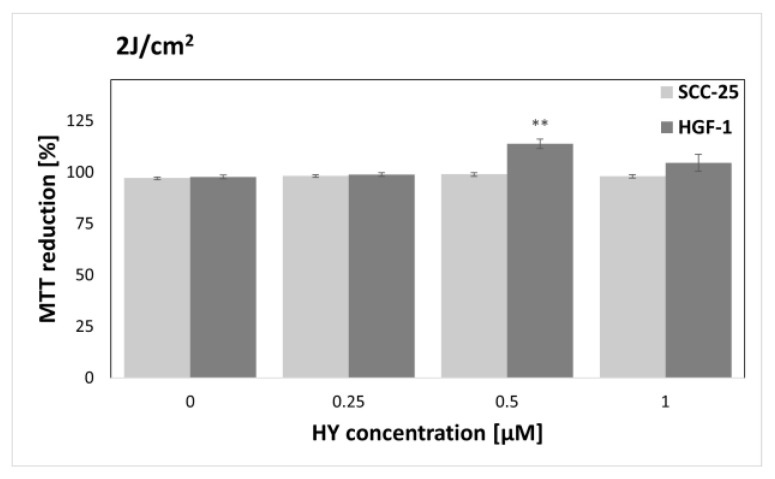
MTT reduction in the SCC-25 and HGF-1 lines for different HY concentrations: 0 μM, 0.25 μM, 0.5 μM, and 1 μM and light dose 2 J/cm^2^. The values represent the means ± SE. ** *p* < 0.05.

**Figure 6:**
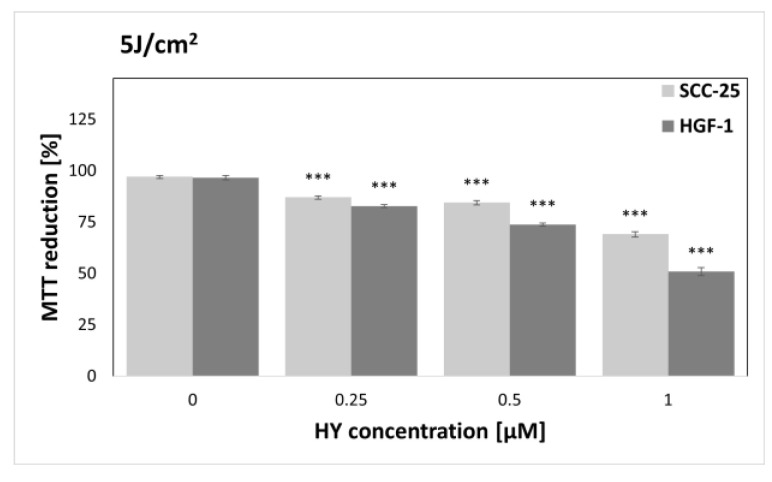
MTT reduction in the SCC-25 and HGF-1 lines for different HY concentrations: 0 μM, 0.25 μM, 0.5 μM, and 1 μM and light dose 5 J/cm^2^. The values represent the means ± SE. *** *p* < 0.01.

**Figure 7:**
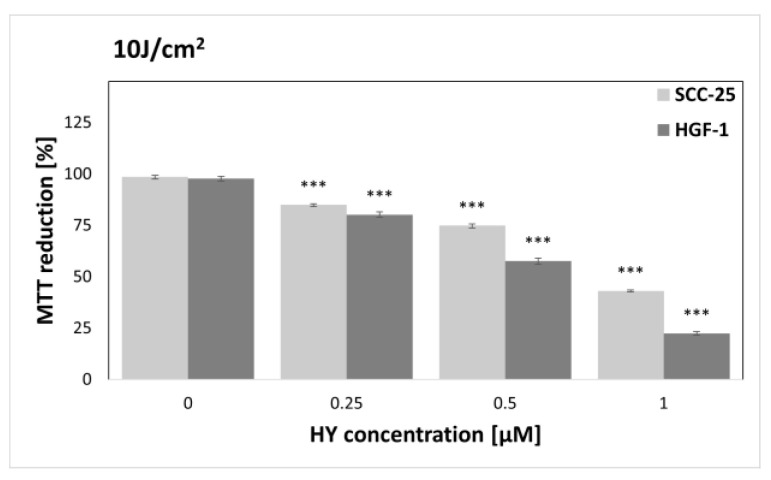
MTT reduction in the SCC-25 and HGF-1 lines for different HY concentrations: 0 μM, 0.25 μM, 0.5 μM, and 1 μM and light dose 10 J/cm^2^. The values represent the means ± SE. *** *p* < 0.01.

**Figure 8:**
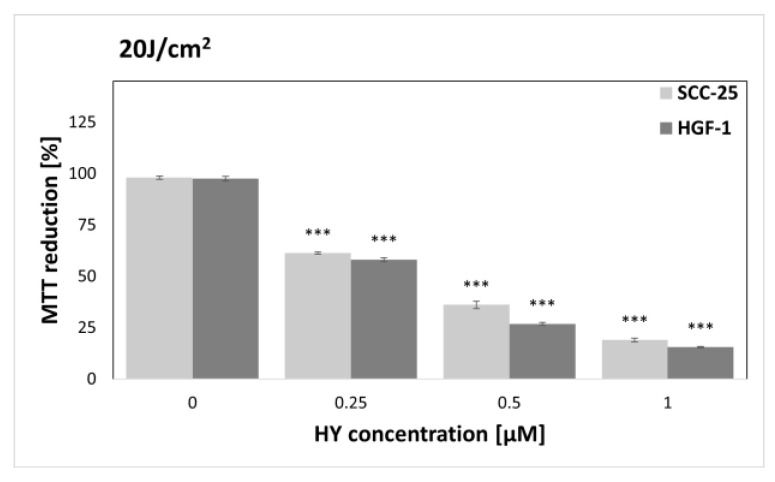
MTT reduction in the SCC-25 and HGF-1 lines for different HY concentrations: 0 μM, 0.25 μM, 0.5 μM, and 1 μM and light dose 20 J/cm^2^. The values represent the means ± SE. *** *p* < 0.01.

The MTT assay was performed to find sublethal doses of PDT. MTT reduction after HY-PDT was compared to a group of native cells, i.e., not exposed to HY and light. In the case of the dark control (Figure 3), no differences in MTT reduction were noted for all doses of HY, while in the case of gingival fibroblasts, the level of MTT reduction decreased at 1 µM HY (MTT reduction = 83.87% ± 0.83%) compared to the native control. Similar results were obtained for a light dose of 1 J/cm^2^ (Figure 4). In the case of the SCC-25 cells, there was no statistically significant difference in the MTT reduction for all HY doses. In turn, in the case of HY 1 µM, there was a decrease in the MTT reduction in the HGF-1 cells (MTT reduction = 91.06% ± 1.62%). In the case of the light dose of 2 J/cm^2^ (Figure 5), no significant decrease in the MTT reduction for both cell groups was observed. However, a stimulating effect was found for HGF-1 at the HY dose of 0.5 µM (MTT reduction = 112.23% ± 3.73%). For the light doses of 5 J/cm^2^, 10 J/cm^2^, and 20 J/cm^2^, an increase in the cytotoxic effect is noticeable with increasing light dose and HY concentration, as shown in Figure 6, Figure 7 and Figure 8. Due to the obtained MTT reduction results, the supernatants of cells treated with concentrations of 0 µM, 0.25 µM, and 0.5 µM for the dark control and light doses of 1 J/cm^2^ and 2 J/ cm^2^ were used for further assays.

### 3.3. Effect of Hypericin-PDT on Secretory Activity: sTNF-R1

**Figure 9:**
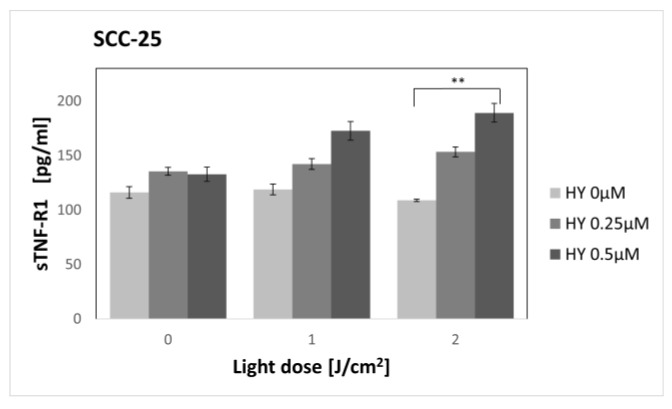
The concentration of sTNF-R1 in the supernatants from oral cancer cell culture SCC-25 line. The values represent the means ± SE. ** *p* < 0.05.

**Figure 10:**
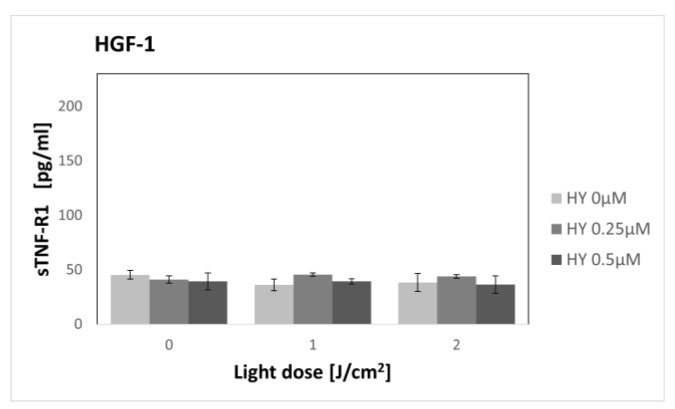
The concentration of sTNF-R1 in the supernatants from gingival fibroblast HGF-1 line. The values represent the means ± SE.

In the case of the SCC-25 cell line (Figure 9), there was no change in the concentration of sTNF-R1 for different doses of HY in the dark control. For the 0 µM HY dose, the level was 116.14 pg/mL ± 5.46. After applying PDT, an increase in the concentration of sTNF-R1 was seen in the supernatants of the SCC-25 cells. Statistical significance was demonstrated for a light dose of 2 J/cm^2^ between HY 0 µM where the sTNF-R1 level was 108.94 pg/mL ± 0.99 and HY 0.5 µM with a level of 189.19 pg/mL ± 2.6. PDT had no effect on the concentration of sTNF-R1 in the HGF-1 supernatants (Figure 10). For the control group, the concentration value was 45.59 pg/mL ± 1.95. Statistically significant differences in the sTNF-R1 secretion were found between the corresponding groups of the SCC-25 and HGF-1 cells, with higher values for cancer cells.

### 3.4. Effect of Hypericin-PDT on Secretory Activity: sTNF-2

**Figure 11:**
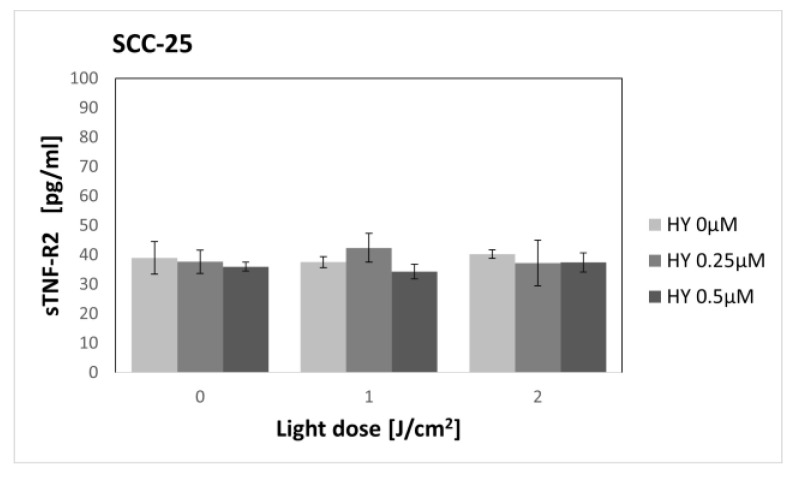
The concentration of sTNF-R2 in the supernatants from the oral cancer cell culture SCC-25 line. The values represent the means ± SE.

**Figure 12:**
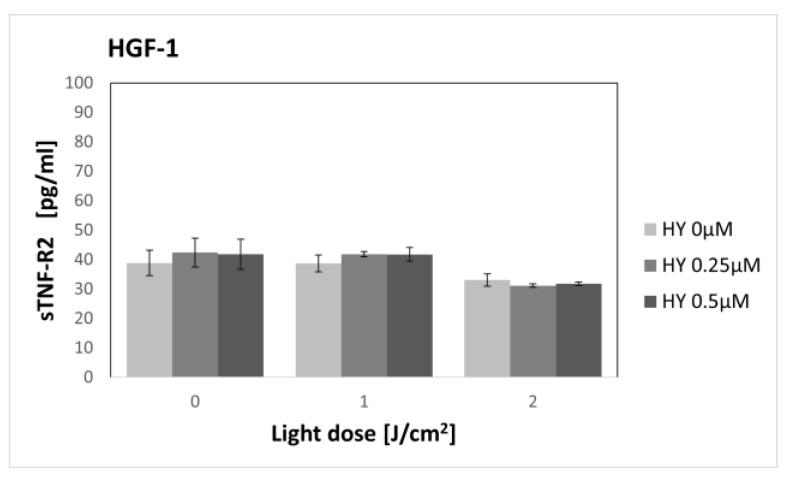
The concentration of sTNF-R2 in the supernatants from the gingival fibroblast HGF-1 line. The values represent the means ± SE.

HY-PDT did not affect the secretion of sTNF-R2 in both the SCC-25 and HGF-1 lines (Figure 11 and Figure 12). In the case of cancer cells, the level of sTNF-R2 for the dark control without PS was 39.01 pg/mL ± 5.51, and for gingival fibroblasts in the corresponding group, it was 38.87 pg/mL ± 4.36.

## 4. Discussion

PDT can be successfully used in precancerous and OSCC lesions [19,20,21]. Our experiment used hypericin as a photosensitizer and a non-coherent VIS light source. A previous in vitro cancer cell study used a similar methodology [40].

HY is a PS of natural origin with hydrophobic properties. Its insolubility can be overcome by drug carriers or conjugates [41,42]. Besic Gyenge et al. showed in their studies that HY mainly accumulated in HNSCC cells in the outer cell membrane, nuclear membranes, trans-Golgi network, and perinuclear space. They also found the cytotoxic effect of HY in the dark at a concentration of 1.19 µM after five-hour incubation [43]. In our study, HY at a concentration of 1 µM showed a cytotoxic effect after two-hour incubation against a gingival fibroblast line but not against oral cancer cells (Figure 3). In addition, Bublik et al. found the accumulation of HY in membranes and the perinuclear space in HNSCC cells [44]. After irradiation, they observed the rupture of cell membranes. They saw a linear increase in cytotoxicity with an increase in HY concentration for the concentrations between 0.1 µM and 2 µM, reflected in the phototoxicity results we observed. Sharma et al. showed more effective effects of HY-PDT after two treatments, one day apart, on the skin SCC cells. The cytotoxic effect after a single PDT was achieved at a concentration of 5 µM HY and a light dose of 1 J/cm^2^, while the second PDT caused a significant decrease in cell viability already at 3 µM HY concentration and the same light dose, which was related to the level of reactive oxygen species. They also found that necrosis was the main route of cell death [45]. Ali et al. found in their study that HY-PDT caused an increased expression of CD95/CD95L in the HNSCC cells, and cell death was dependent on CD95 signaling and led to apoptosis [46]. HY, apart from PDT, can be used in the photodiagnostics of neoplastic and precancerous lesions of the oral cavity due to the selective uptake of the dye by abnormal cells [47].

In the case of HGF-1 cells irradiated with a dose of 2 J/cm^2^ and pre-incubated with 0.5 µM HY, an increase in MTT reduction was demonstrated (Figure 5). That isolated result was not observed at other doses and with SCC-25 cells. A possible source of such an image is the stimulating effect of low doses of light. Etemadi et al. demonstrated the stimulating effect of light in low doses on healthy gingival fibroblasts 24 h after irradiation [48].

The presence and behavior of the persistent cells may influence the recurrence of a neoplastic lesion or the formation of metastases or induce inflammation. Therefore, it is essential to find the sublethal dose of PDT and its effect on the activity of cancer cells. Kaleta-Richter et al. studied the effect of HY-PDT on the secretory activity of colorectal cancer (CRC) cells. In the study, they found an increase in the secretion of IL-8 by the more malignant line SW620, while in the case of the lower-grade SW480, a decrease in the secretion of this cytokine was observed. They found no impact on Il-10 levels [40]. Hu et al. showed in their studies that HY-PDT inhibited CRC cell proliferation, induced cell cycle arrest in the S phase, and caused cell apoptosis. The researchers found an increase in the expression of Bax and a decrease in the expression of Bcl-2, followed by an increase in the expression of cleaved caspase-9, cleaved caspase-3, and cleaved PARP, leading to apoptosis [49]. In addition, PDT, with the commonly used PS precursor 5-aminolevulinic acid, had an immunomodulatory effect on CRC cells [33,34,35,36].

By determining the secretion of sTNF-R1 and sTNF-R2, we wanted to evaluate, in addition to the direct cytotoxicity of PDT, the immunomodulatory effect. Soluble forms of receptors may weaken the effect of TNF-α by competitively binding to membrane receptors, or they may prolong its activity by stabilizing it and protecting it against degeneration [27]. TNF-α is a cytokine that creates a proinflammatory microenvironment. A characteristic of PDT is the generation of inflammation, which is involved in the long-term control of tumor growth. Highly inflammatory PDT regimens have been reported to induce significant acute inflammation characterized by increased expression of proinflammatory cytokines including TNF-a, IL-1b and IL-6, adhesion molecules, E-selectin, and ICAM-1. They also cause infiltration of the primary tumor and tumor-draining lymph nodes (TDLNs) by leukocytes. PDT induces antitumor immunity; a study in mice showed the formation of immune memory. Cell transfer from TDLNs to naive hosts conferred immunity on subsequent tumor challenge [50]. Regression of untreated distant tumors by cell-mediated immunity has been reported after the use of PDT in multifocal angiosarcoma of the head and neck [51].

In our study, the highest dose not causing a cytotoxic effect was 0.5 µM HY with a light dose of 2 J/cm^2^. Therefore, sTNF-R1 and sTNF-R2 levels were determined for sublethal doses. To our knowledge, this is the first study to assess the effect of PDT on the secretion of soluble TNF-α receptors. We found an increase in sTNF-R1 secretion by OSCC after sublethal doses of PDT, while PDT had no effect on sTNF-R1 secretion by healthy gingival fibroblasts. We found no change in the production of sTNF-R2 by both cell lines, regardless of the dose of PDT used.

Stracher, in a study on a mouse animal model, showed the important role of TNF-α and its receptors in tumorigenesis. Mice lacking TNF-R1 or TNF-R2 were less likely to develop skin cancer in response to UVB irradiation. Mice lacking TNF-R1 showed no significant influx of inflammatory cells into the chronically irradiated area, while TNF-R2 KO mice showed a normal influx of inflammatory cells [52]. Tanaka et al. found that highly metastatic OSCC cells secreted a greater amount of TNF-α and were characterized by a greater expression of the membrane form of the TNF-R1 receptor. As a result, the activity of NF-κB was amplified, which led to increased secretion of metalloproteinase 2 (MMP-2) and MMP-9 and increased invasive activity [53]. Kamińska et al., in a study of the serum of patients with CRC, found a significant increase in sTNF-R1 in the case of intestinal wall infiltration that was strongly correlated with the advancement of the tumor, being a prognostic factor [54]. In addition, in patients with cervical adenocarcinoma, sTNF-R1 showed elevated levels and was a prognostic factor [55]. In turn, Burger et al., in their study of patients with ovarian epithelial malignancy, found that a low risk of cancer progression occurs in cases with high sTNFR-I levels and low sTNFR-II, and a heightened risk of progression occurs in cases with low sTNFR-I levels and high sTNFR-II in serum [56].

## 5. Conclusions

We confirmed the cytotoxic effect of PDT using HY. The cytotoxic effect was dependent on the dose of HY and light. A sublethal dose of HY affects the secretory activity of oral cancer cells. PDT did not affect sTNF-R1 production by HGF-1 cells. No effect of PDT on the sTNF-R2 secretion by either cell line was demonstrated. Increased secretion of sTNF-R1 was found after sublethal doses of PDT. Due to the multiple effects of TNF-α and its soluble receptors, which can stabilize and prolong the action of TNF-α and be an antagonist, further research is necessary. It is important to confirm our observations in further preclinical studies on an animal model, preferably in clinical trials.

## Data Availability

Department of Orthodontics, Faculty of Medical Sciences in Zabrze, Medical University of Silesia, Katowice, Poland.
